# Open hands, large numbers: manual gestures influence random number generation

**DOI:** 10.1007/s00426-025-02085-5

**Published:** 2025-02-19

**Authors:** Caterina Villani, Glenn De Muynck, Anna M. Borghi, Luisa Lugli, Bodo Winter

**Affiliations:** 1 Department of Modern Languages, Literatures, and Cultures, University of Bologna, Via Cartoleria 5, 40124 Bologna, Italy; 2 Department of Philosophy, University of Bologna, Via Zamboni 38, 40126 Bologna, Italy; 3 Department of Dynamic and Clinical Psychology, and Health Studies, Sapienza University of Rome, Via Degli Apuli 1, 00185 Rome, Italy; 4 Institute of Cognitive Sciences and Technologies, National Research Council, Via Romagnosi 18A, 00196 Rome, Italy; 5 Linguistics & Communication, University of Birmingham, Birmingham, UK

## Abstract

Several studies suggest that numerical cognition interacts with spatial cognition. Here, we explored spatial-numerical associations through the lens of manual gestures. We asked English and Italian participants to generate ‘random’ sequences of numbers while simultaneously moving the hands outwards, away from the torso, or inwards, towards the mid center of the body. These manual gestures were modeled after gestures that are common in naturally occurring numerical discourse, such as when people talk about “huge numbers” or “tiny numbers.” Results showed that in both participant groups, outwards movements coincided with relatively larger numbers compared to inwards movements, for which generated numbers were smaller. This effect was small in magnitude. We also explored individual differences and found that self-reported numeracy as well as levels of gesture production and perception modulated the effect of our gesture manipulation very little, if at all.

## Introduction

We often use our hands to communicate quantities. We count with our fingers, hold our hands wide open to refer to a large group of people, or pinch our fingers together when asking a waiter to serve only a small amount of water. All these everyday gestures reflect how much numerical thinking and communication involve spatial representations, such as mapping precise numerical values to individual fingers, or expressing quantity via the space spanned between the hands.

Research investigating number processing has consistently suggested that there is a mental connection between our conceptualization of numerical quantities and spatial information (for reviews, see Winter et al., 2015a, 2015b; Walsh, 2003), which is often seen in the context of embodied numerical cognition (e.g., Fischer, 2012). This connection has been documented in various spatial-numerical associations, where quantities are mapped along spatial directions, or are conceived as having spatial extensions, where larger numbers occupy more space (for a taxonomy of spatial-numerical associations, see Cipora et al., 2020). Here, we focus on spatial-numerical associations involving size. Classic behavioral experiments show that people are quicker to correctly judge which of two numbers is greater when the larger number is presented in a larger font and when the relatively smaller number is presented in a relatively smaller font (Henik & Tzelgov, 1982). Similarly, when asked to judge which of two quantities of dots is greater, people are quicker when the more numerous display occupies more space on the screen (Hurewitz et al., 2006). These findings demonstrate physical size cognitively interacts with numerical magnitude, where people associate larger quantities with a larger area or volume, and smaller quantities with a smaller area or volume.

Further studies have linked these size-based spatial-numerical associations to action-related processes that involve the hands. Various strands of evidence show that numerical magnitude is related to specific hand shapes used to interact with objects of different sizes. For example, in a parity judgment task, Lindemann et al. (2007) found that participants are faster at initiating a precision grip (index finger and thumb together, as if holding a small pellet) in response to smaller numbers, and initiating a power grip (all fingers grasp, as if holding a pipe) in response to larger numbers. In a similar experiment, Andres et al. (2008) found that when participants reach for blocks of the same size with different numbers written on them, they spontaneously widen their grip aperture for relatively larger compared to smaller numbers. Even simply observing manipulable objects influences number processing in parity tasks, with facilitation for smaller over larger numbers when viewing graspable objects compared to ungraspable objects (e.g., Ranzini et al., 2011; Badets & Pesenti, 2011; for evidence on action execution, see Namdar & Ganel, 2017). Similarly, the processing of surface characteristics of digit stimuli, such as color, can automatically prime grasping gestures, with small numerical values facilitating precision grips, and large numerical values facilitating power grips (Moretto & Di Pellegrino, 2008). Recent studies using the bimanual grip force recording technique during numerical processing tasks have found that participants tend to map number magnitude on grip width, with smaller number led to a relative increase of the holding force in the left hand, while larger numbers increased the relative right-hand holding force (Miklashevsky, 2022; Miklashevsky et al., 2021). Taken together, these findings suggest that the link between physical size and numerical cognition extends to motor actions performed with the hand.

The evidence for spatial-numerical associations, and their relevance for manual actions, is not limited to experiments in laboratory settings, it also consistently occurs in spontaneous verbal communication. Recent observational studies confirm that size-based gestures are prominent in numerical discourse (Alcaraz-Carrión & Valenzuela , 2022; Winter et al., 2013; Woodin et al., 2020). Woodin et al. (2020) conducted a large-scale, quantitative investigation of TV news casts, focusing on manual gestures that were produced alongside the expressions “tiny/small/large/huge number(s)”. Results showed that, just as these verbal expressions use spatial words to describe quantity (Lakoff & Johnson 1980, 2008; Winter et al., 2015a, 2015b), the size implied by a speaker’s gesture mimicked numerical quantity. For instance, when talking about a “huge number”, newscasters and politicians frequently performed gestures in which their hands moved outwards, away from the mid center of their body, with flat, open palms facing one another. On the other hand, when referring to “tiny numbers”, they frequently performed “precision grip” type gestures where the forefinger and thumb approach or touch each other; or they performed inwards movements where both hands move closer to each other in front of the body. Such gestures are surprisingly common: Woodin et al. (2020) report that speakers gesture about 80% of the time when they use spatial language to refer to numbers. Such observational studies suggest that the spatial-numerical associations that have previously only been established under controlled experimental conditions in the laboratory, are active and alive in naturalistic discourse, shaping the way people use their hands when talking about quantities (see also Alcaraz-Carrión & Valenzuela, 2022).

Although Woodin et al. (2020) observed correlations between gestures and spatial language used in numerical contexts (e.g., “huge number”), this study does not conclusively show that spatial gestures interact with numerical cognition. First, gestures accompanying words like “huge” could simply reflect conventions associated with spatial language, without involving genuine mappings between space and number (see arguments in Bouissac, 2008). That is, an outwards-gesture produced alongside the expression “huge number” could simply result from lexical priming of the word “huge”, and not from an underlying conceptualization of number in terms of size. For this reason, we want to explore the impact of size-based gestures on pure number processing, unfettered by intervening spatial language.

One widely used task to probe numerical representations as well as spatial-numerical associations is the random number generation task (RNG). In this task, participants are instructed to generate numbers as “randomly” as possible (see Nickerson, 2002, for a critique of the concept of randomness in this task). There are many different versions of this task. Researchers interested in numerical cognition have looked at how number generation behavior differs as a function of concomitant body movements (Hartmann et al., 2014; Loetscher et al., 2008, 2010; Shaki & Fischer, 2014; Winter & Matlock, 2013). So far, these studies have largely looked at directional spatial-numerical associations, such as the left-to-right going mental number line (Dehaene et al., 1993; Wood et al., 2008). For example, when people look to the left or to the right to the beat of a metronome, they produce smaller or larger numbers, respectively, as if numbers were mentally represented on a mental number line (Loetscher et al., 2008; see also Loetscher et al., 2010).

In the present study, we bring the gestures observed by Woodin et al. (2020) back into the lab, using them to investigate whether manual gestures modeled after naturally occurring numerical gestures can modulate participants’ performance on a random number generation task. Building on this observational work, we focused on inwards and outwards movements that people typically produce in association with larger and smaller numerical quantities, respectively. During the task, participants will alternately perform inwards movements (hands moving toward each other, as if showing a small object to an interlocutor) and outwards movements (hands moving away from each other, as if showing a large object), while producing numbers ranging from 1 to 30 (1 and 30 included) to the beat of a metronome. In line with previous studies, we expect a directional influence of gestures on RNG. Specifically, we expect outwards gestures to lead to larger magnitudes than inwards gestures, which conversely should be associated with overall smaller magnitudes in this task. Importantly, in contrast to previous studies investigating the impact of body and eye movements on RNG behavior, our gestures are ecologically motivated: we have evidence from observational studies that these gestures actually occur. There is no such evidence for the head turns investigated by Loetscher et al. (2010), for example. Thus, we investigate a behavior that actually occurs in spontaneous discourse when people communicate numerical concepts.

We also use our RNG task to probe the link between spatial-numerical associations and math achievement. Research on embodied numerical cognition has pointed out that gestures are one powerful tool for bolstering mathematical learning (Goldin-Meadow, 2003; Goldin-Meadow et al., 2001; Marghetis & Núñez, 2013; Weisberg & Newcombe, 2017). Students and teachers often use spatial gestures to convey abstract numerical concepts (Goldin-Meadow, 2003; Núñez, 2004). Children learn new math concepts better when instructions include corresponding gestures compared to when they do not (Cook & Goldin-Meadow, 2006; Cook et al., 2012; Nemirovsky et al., 2012). Although spatial abilities are considered crucial for math skill development (Hawes & Ansari, 2020), the link between spatial-numerical associations and math abilities remains debated due to numerous null results and conflicting evidence in children and adults (Cipora et al., 2020). The literature on finger gnosia (the ability to recognize and distinguish one’s own fingers, both visually and through touch) shows that finger representations do, at least weakly, correlate with future success in mathematics education (Costa et al., 2011; Fischer et al., 2022; Noël, 2005; Penner-Wilger & Anderson, 2013; Wasner et al., 2016). In adults however, the evidence for the functional relevance of spatial-numerical associations is more mixed. Cipora and colleagues (Cipora & Nuerk, 2013; Cipora et al., 2015, 2018a, 2018b) show that numeracy does not correlate with the strength of horizontal spatial-numerical associations. In another study, Cipora et al. (2016) show that mathematicians might even have weaker spatial-numerical associations than non-mathematicians.

Here, we are interested in seeing whether people with higher or lower numeracy, assessed through the Subjective Numeracy Scale (SNS) (Fagerlin et al., 2007), show stronger or weaker evidence for spatial-numerical associations in our RNG task. It is conceivable that people with lower numeracy have less developed numerical representations that are more susceptible to gestural influence; conversely, if spatial-numerical associations *help* in numerical cognition, we might expect that people with higher numeracy show stronger effects of gesture. We will also use the Brief Assessment of Gesture (BAG) scale (Nagels et al., 2015) to see whether people’s self-reported gestural behavior interacts with our findings: it is plausible that people who rely more on gestures in their everyday behavior would also show stronger gestural effects in our task.

Finally, we further explore variations in gesture behaviors at the cultural and linguistic level, involving participants of two cultures, Italian and English, that differ in how extensive their vocabulary of gestural forms is (see Cattani et al., 2019; Graziano & Gullberg, 2024). Italian speakers are often perceived as using gestures more frequently and with larger, more expressive movements than speakers of other languages, such as Spanish and English (Sekine et al., 2015). Similarly, Italian children gesture more frequently than their American and French peers (Iverson et al., 2008; Capirci et al., 2010; Colletta et al., 2015; see also Cattani et al., 2019). However, this pattern has not been replicated when Italians are compared with other cultural groups, such as Japanese (Pettenati et al., 2012). By involving Italian and English-speakers participants, we aim to explore whether the effect is stronger in cultures with a high frequency of spontaneous gestures and to increase the generalizability of the effect if no substantial differences were observed between the two cultural groups. Additionally, looking at how our results correlate with individual difference measures (SNS, BAG, cultural/linguistic backgrounds), we add to the growing literature on individual differences in embodied cognition (for a recent discussion see Miklashevsky & Jeglinski-Mende, 2024; Ibáñez et al., 2023).

## Methods

### Participants

Our pre-registered sample size justification aimed for at least 60 participants in total (30 for each language group: English and Italian). This number was partially motivated through feasibility constraints, with 30 being what can realistically be achieved in terms of data collection in our respective departments responsible for data collection. We did not conduct a formal power analysis due to the novelty of our tasks: published RNG studies used very different bodily manipulations, and past studies using bodily manipulations did not consistently report effect size, making it hard to gauge what effect sizes were reasonable. We had reason to expect that our effect was going to be larger than those observed in previous RNG studies since our movements were (1) ecologically motivated, i.e., actually attested in numerical discourse when people use spatial language (Woodin et al., 2020), and (2), by involving both arms held widely apart or closely together, they were also larger movements than those featuring in previous studies. For example, Loetscher et al., (2008, *n* = 40) and Winter and Matlock (2013, *n* = 65) used comparatively smaller head movements, and Loetscher et al., (2010, *n* = 30) involved eye movements. The number 60 was thus pragmatically motivated to be at least as large as previous RNG studies that successfully obtained effects which we have reason to believe were smaller than ours.

For reasons involving counter-balancing detailed below (see Section "Deviations from pre-registration"), we decided to deviate from our pre-registration plan and double the sample size by running the study an additional semester. Following Giner-Sorolla et al. (2024), an effect size sensitivity analysis shows that with 120 participants, an effect would have to be larger than Cohen’s *d* = 0.36 to achieve 80% power—our actually observed effect size is in excess of this (*d* = 0.48).

Italian-speaking participants were recruited as volunteers among students and researchers of the Cognitive Psychology course at the Dept. of Philosophy at the University of Bologna. After one participant was excluded because they were not able to perform the task, and another participant was excluded because they were not a native speaker of Italian, the final sample of Italian speakers was 61 participants (40 female, 21 male; mean age: 24.5, range 19–35; 5 left-handers).

English-speaking participants were recruited from undergraduate linguistics and literature students at the Dept. of Linguistics & Communication at the University of Birmingham for partial course completion. After two participants were excluded because they were not native speakers of English and three participants experienced difficulty with the task, the final sample of English speakers was 59 (49 female, 10 male; mean age: 19.6, range 18–23; 5 left-handers).

Ethical approval was provided by the Ethics Committee of the Sapienza University of Rome, Department of Dynamic and Clinical Psychology and Health Studies (Protocol Number: 0001743). All participants signed an informed consent form prior to the beginning of the experiment.

### Procedure

Data collection was carried out simultaneously at the University of Birmingham and the University of Bologna in two different periods: February–March 2023 and February–March 2024.

Before starting the experiment, each participant was asked to sign the informed consent and provide demographic information, i.e. age, education level, gender identity, handedness, and linguistic background. During the experiment, participants sat in a quiet and lit room in front of a video camera. They were asked to call out random numbers between 1 and 30 (1 and 30 included) at the beat of 0.5. Hz (30 bpm) played by an electronic metronome,^1^ while performing two-handed gestures with inwards movements (hands moving toward each other) and outwards movements (hands moving away from each other) on the horizontal axis of their torso. The metronome setup established a rhythm for the random number generation of 2 s per beat. We selected the numerical range of 1 to 30 to align with prior research (Loetscher et al., 2008; Hartmann et al., 2012; Winter & Matlock, 2013).

The experiment consisted of two phases. In the training phase, we first instructed participants to rehearse performing the 10 rhythmic hand movements to the beat for 5 inwards and 5 outwards trials. Then we introduced the random number generation component, asking participants to produce 10 numbers “as random as possible”, and then participants performed 10 practice trials where they produced random numbers and performed inward-outward movement simultaneously. Instructions (pre-registered in a repository: 10.17605/OSF.IO/J7EU5) highlighted that randomness means that each number has an equal probability and that each number is independent of the preceding one. We asked participants not to call out the number *while* performing the movement but when the hands are stationary in the positions of the horizontal axis. This instruction was intended to help participants to synchronize rhythm, movement, and number production while reducing variability in the timing of number production across participants due to differences in the extent of their inward and outward hand gestures. Timing both number production and the movement’s end position to the sound of a beat facilitates the tasks. To avoid bias in the instructions, the experimenter never mentioned any numbers or spatial language to describe numbers (e.g., “small number”, “large number”). Following previous studies (Loetscher et al., 2008; Winter & Matlock, 2013), we asked participants to close their eyes while performing the task.

**Fig. 1:**
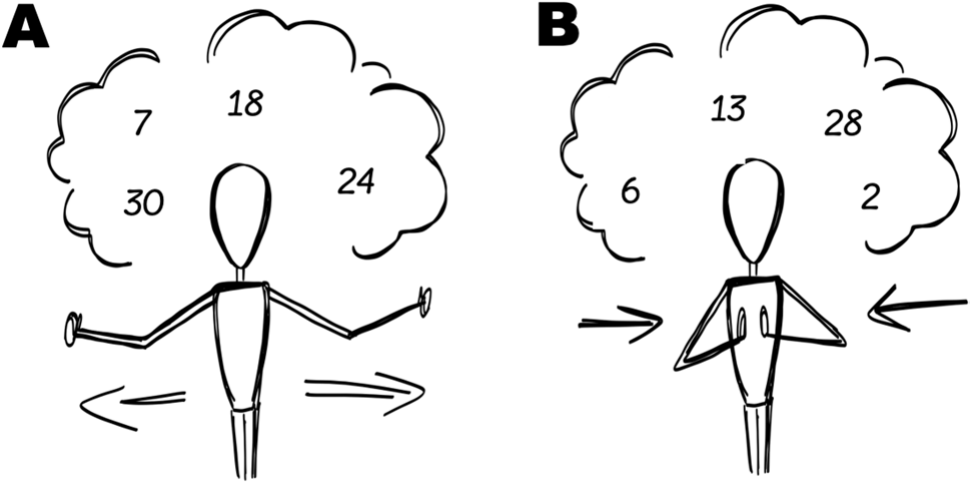
Sample **a** outwards and **b** inwards gestures. Drawing by Alexandra Lorson

In the main experimental phase, participants were asked to generate 80 numbers while performing the hand movements on the beat (see Fig. 1 for schematic depictions of these gestures). The starting of the hands i.e., whether the first trial is moving outwards, or inwards, was counterbalanced between participants (see below).

The verbal responses were recorded manually by the experimenter on paper sheets and subsequently entered into a spreadsheet. In some cases, participants interrupted themselves and had to start again. If this happened, a row in the paper sheet was marked as missing value. The paper sheet listed more than 80 trials and experimenters were instructed to top up each experimental session with more trials in case there were any self-interruptions so as to aim for 80 trials per participant even in the case of missing values. After the experiment, participants completed two surveys: the Subjective Numeracy Scale (SNS) (Fagerlin et al., 2007), consisting of 8 items assessing self-perceived mathematical ability and preference for numerical versus prose information, and the Brief Assessment of Gesture (BAG) scale (Nagels et al., 2015), comprising 12 statements on spontaneous gesture production and perception. The entire experiment lasted approximately 20 min.

### Statistical analysis

All data and analysis code is available on the following Open Science Framework repository: https://osf.io/9esjz/.

We used R version 4.3.0 (R Core Team, 2019) and tidyverse 2.0.0 (Wickham et al., 2019) for data processing, analysis, and visualization. The package brms 2.21.0 (Bürkner, 2017) was used to fit Bayesian models. The package effsize 0.8.1 (Torchiano, 2019) was used to compute Cohen’s *d*. The package pwr version 1.3.0 was used for effect size sensitivity analysis (reported in Section “Participants”) (Champely, 2020).

Our key research question is whether the magnitude of the numbers generated by participants is influenced by the distinction between outwards and inwards trials. Magnitude can be captured absolutely, i.e., whether numbers are overall larger on outwards gestures and smaller on inward gestures; it can also be captured relatively, i.e., whether the number produced on an outwards trial is larger than the previous one (cf. Winter & Matlock, 2013). We analyze the data from both perspectives, which requires different kinds of statistical models as absolute and relative magnitudes have different distributional properties.

For absolute magnitudes, we are using a mixed beta-binomial regression model. In most previous papers on random number generation, people use approaches such as t-tests, ANOVAs, or standard linear mixed effects models on RNG data. This is problematic as we know on a priori grounds that it is impossible for these approaches to capture the distribution of RNG data, which is *discrete* (i.e., numbers can only be “1”, “2”, etc. but not “1.3”), and *bounded* (i.e., numbers cannot exceed the range 30). Both of these distributional properties are at odds with t-tests and similar such approaches, which means that previous approaches of analyzing RNG data have failed to model an appropriate data-generating process (indeed, posterior predictive simulations of models with underlying Gaussian processes show that these approaches grossly misfit RNG data). The beta-binomial distribution is a discrete bounded distribution, i.e., in our case, it models the probability of any of the numbers 1–30 occurring. So, we assumed absolute magnitudes to be generated by a beta-binomial process (logit link), with the model specified to have the fixed effects gesture (outwards versus inwards), language (Italian versus English), and the gesture * language interaction. For random effects, this model includes random intercepts for participants, as well as by-participant varying slopes for the effect of gesture.

The second approach looks at relative magnitudes, for ease of discussion and analysis, dichotomized into a simple variable that codes for whether the number at trial *t* + 1 is higher or lower than the number at trial *t*. Because the response variable is binary, a standard mixed effects logistic regression model with Bernoulli data-generating process is appropriate. This model included the same fixed and random effects as the previous analysis.

As both the beta-binomial model of absolute magnitudes and the logistic regression model of relative changes include interactions, we sum-coded the language and gesture predictors (language: -1 English, + 1 Italian; gesture: -1 inwards, + 1 outwards) to facilitate the interpretation of main effects in the presence of interactions. We used the default priors specified by brms except for hand-specifying a weakly informative prior on the fixed effects coefficients of the logistic regression model, a Cauchy prior with scale 2.5 (see Gelman et al., 2008). Models were estimated in Stan using Markov Chain Monte Carlo (MCMC) simulation with 4 chains for 5,000 iterations (3,000 warm-up samples excluded), which leaves 8,000 posterior samples used for inference.

### Deviations from pre-registration

All methods and analyses have been pre-registered (https://doi.org/10.17605/OSF.IO/J7EU5). In this section, we want to draw attention to aspects of data collection and analysis that deviate from our original plan, as specified in the pre-registration document.

*Differences in sample.* Our pre-registration document failed to specify a variable that we realized later was going to be of importance: the starting position of the hands, i.e., whether the first trial is moving outwards, or inwards. After piloting the experiment amongst ourselves, we noticed that it is easier to start the first trial on an outwards movement. To make this already challenging task easier for our participants, we instructed them to assume this starting position on the first trial. After collecting the data, however, we noticed that the numbers 1 and 30 were quite common on the first trial, which was likely due to the fact that these numbers were mentioned last in the instructions (“…generate a number from 1 to 30…”) (for exact figures, see results, Section "Numbers driving the effect"). This induces a bias in the direction of our hypothesis as the maximum number (30) will be primed and coincide with an outward gesture. As we were concerned that this would unduly bias our results in favor of our hypothesis, we decided to double the data set size from what was specified in the pre-registration to collect data of equal sample size from the other starting position (first trial inward). This explains why we have collected more data than specified in the pre-registration, and why there have been two bouts of data collection.

It should be stressed that our decision to collect additional data was in no way contingent on having found reliable evidence for an effect (Simmons et al., 2011). In fact, the opposite is the case: the first bout of data collection already showed the effect in question, but additional data was collected to ensure that the results we report below did not simply stem from a bias induced by the starting position. The pre-registration also specifies that we originally intended to perform the same task with mathematics students. Due to the increased data collection efforts of the main study, we had to drop this element of our study for feasibility reasons.

*Differences in analysis*. Upon analyzing the final data, we realized that additional data exclusions were necessary that were not foreseen in our pre-registration as the issue did not come up during piloting. Specifically, we had to exclude numbers that were outside of the instructed range (e.g., “0”, “31”). The pre-registration also failed to specify an important detail that surfaced in the analysis of relative differences in the final data: As described in Section "Procedure", when participants initiated trials after a restart, we indicated this as missing data for the beat. This meant that relative differences could not be computed adjacent to missing values, an aspect of our data analysis not reflected in the pre-registration.

## Results

### Absolute magnitude

On outwards trials, the grand average of all numbers for the entire dataset is 14.8, compared to 14.0 on inwards trials. English and Italian participants both showed the same pattern to similar degree (English: outwards: 14.5, inwards: 13.6; Italian: outwards: 15.1, inwards: 14.4). To assess how consistent this effect was across the cohort, we computed average difference scores (outwards – inwards) separately for each participant. The sign of these differences was congruent with our hypothesis (larger numbers for outwards trials) for 78 participants, 65% of the total. For 42 participants, 35%, the sign was incongruent with our hypothesis. This coarse measure of consistency does not, however, take inter-trial variability within each participant into account, and is thus void of any inferential uncertainty about each participant’s mean. Following Roth et al. (2024), we computed 90% credible intervals for each participant using the main model described in Section "Statistical analysis", but separately for each participant (and hence omitting the participant-level random effects). For only 16 out of the 120 participants (13.3%) did these 90% credible intervals exclude zero in the direction of the predicted effect. Only 4 participants (3.3%) showed an effect where the 90% credible interval excluded zero in the opposite direction, i.e., these few participants ‘reliably’ produced larger numbers for inwards gestures.

**Fig. 2:**
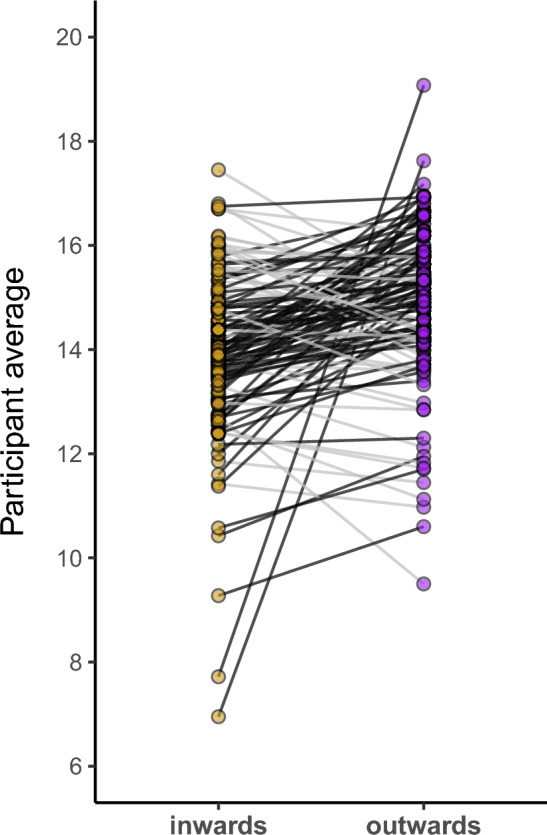
Pair plot showing average number magnitudes for inwards trials (yellow) and outwards trials (purple); each line represents one participant, with line color indicating whether each participant’s effect was congruent (black) or incongruent (gray) with our hypothesis

Figure 2 shows a pair plot of each participant’s averages for outwards and inwards trials. Each line represents a participant, with line color indicating whether the sign of each participant’s effect was congruent (black) or incongruent (gray) with our hypothesis. As can be seen, two participants with very large effects stood out from the rest, the majority of which only showed slightly higher averages for outwards gesture trials, compared to inwards trials. In terms of standardized effect size, Cohen’s *d* (paired) computed on these average differences indicated a “small” effect size of outwards versus inwards gesture, Cohen’s *d* = 0.48 (95% confidence interval [0.24, 0.72]).

**Fig. 3:**
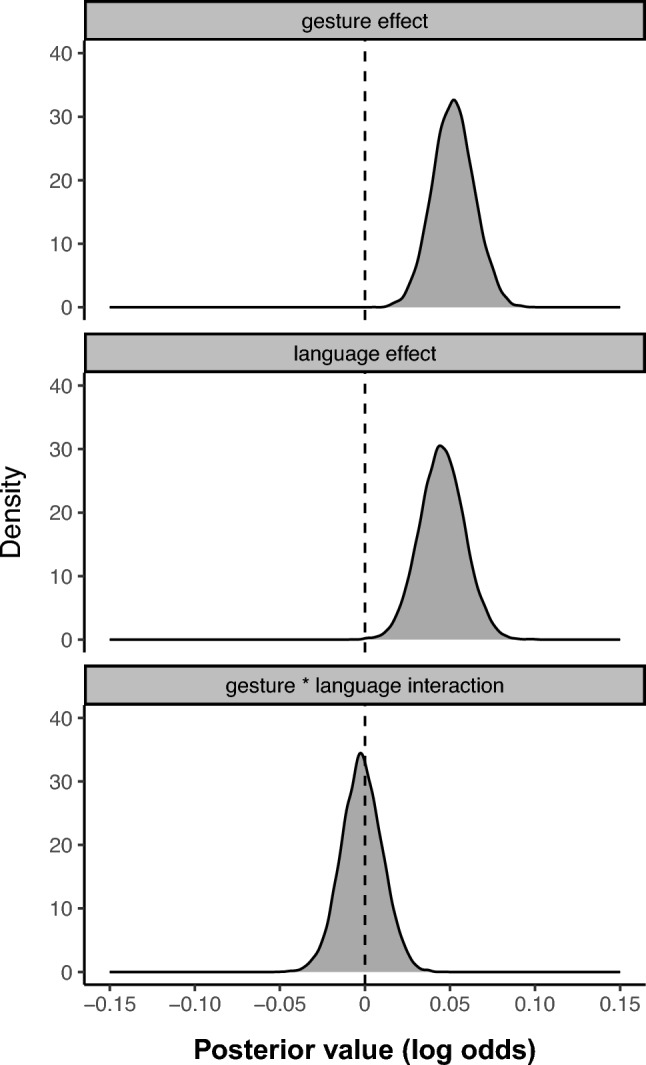
Posterior distributions for the three fixed effects in the main model (mixed beta-binomial regression on absolute magnitudes) that includes the gesture by language interaction

To model uncertainty for these results^2^ and as described in Section "Statistical analysis", we fitted a mixed beta-binomial model with fixed effects gesture (outwards versus inwards), language (Italian versus English), and the interaction of the two, as well as a random effect for participant, including by-participant varying gesture slopes to quantify participant variation in the effect. Figure 3 shows the posterior distributions for each of the three fixed effects. As can be seen, the posterior distributions of both the gesture and the language effect did not substantially overlap with zero, but the distribution of the interaction effect was centered at zero (posterior log odd mean of interaction ~ 0.0, 95% credible interval CrI: [-0.03, + 0.02]). The absence of any indication of an interaction between gesture and language group suggests that the gesture effect was similar for both Italian and English participants. The gesture effect was positive (posterior mean = + 0.05), with a 95% credible interval that excluded zero (CrI: [+ 0.03, + 0.08]), thus indicating high certainty that produced numbers are on average larger for outwards trials. The posterior probability of this group-level effect being of the same sign (outwards > inwards) was 1.0, i.e., 100% of all posterior estimates exceed zero.

We also found an unexpected language effect: the numbers generated by Italian speakers (*M* = 14.7) were slightly higher than those by English speakers (*M* = 14.1). In the model, this effect was of similar magnitude to the gesture effect (posterior mean = + 0.05, 95% CrI: [+ 0.02, + 0.07]), and was also associated with a high posterior probability of being of the same sign (*p* = 1.0). The probability of the expected gesture effect being stronger than the unexpected language effect is only *p* = 0.68, i.e., we cannot be certain of there being a difference between these two fixed effects. Together with the relatively small Cohen’s *d* reported above, this paints a picture of a relatively weak effect, with the gesture coefficient only being 1.15 times larger than the language coefficient.

Additionally, we also assessed the effect of starting position, for which we took the main model with a fixed effect of gesture (outwards versus inwards) and added a fixed effect of starting position (outwards first, inwards first), including the interaction of these two terms (both fixed effects were sum-coded to aid the interpretation of main effects in the presence of interactions). Participants whose first trial was outwards produced overall higher numbers (*M* = 14.6) than people who started on inwards gestures (*M* = 14.2). This effect (log odd coefficient: + 0.03) was associated with a 95% credible interval that barely overlapped with zero (95% CrI: [< 0.00, + 0.05]), resulting in a relatively high probability of being of the same sign (*p* = 0.97). There was, however, no indication of a reliable interaction between starting position and the gesture effect (– 0.01, 95% CrI: [– 0.03, + 0.02], probability of same sign *p* = 0.69). Importantly, this model controlling for starting position also showed a main effect of gesture (logit estimate: + 0.05) whose 95% credible interval also excludes zero (95% CrI: [+ 0.03, + 0.08], probability of same sign *p* = 1.0). These specific results substantially reduces the concern that the starting position of the first bout of data collection may have unduly affected our results (see Section "Deviations from pre-registration").

### Relative differences

On outwards trials, 60.8% of all generated numbers were higher than the previous one for outwards trials, compared to 54.6% for inwards trials.

**Fig. 4:**
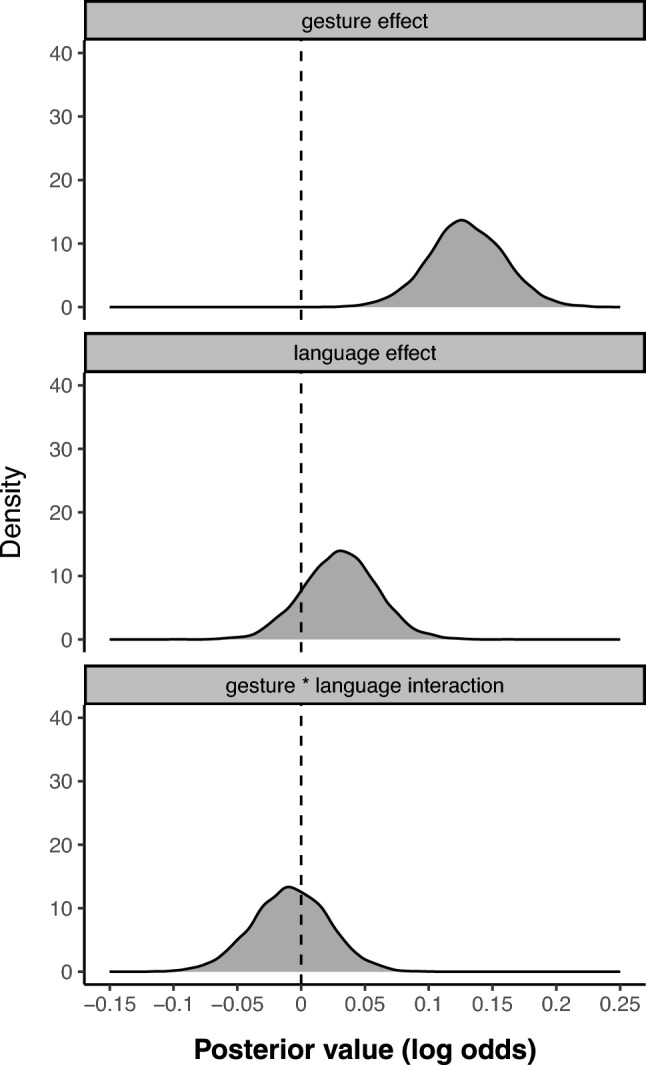
Posterior distributions for the three fixed effects in the logistic regression model (relative differences) that includes the gesture by language interaction

As described in Section "Statistical analysis", we fitted a mixed logistic regression model on a dependent variable coding for whether a given trial was higher or lower than the previous one. This model had the same fixed effects (gesture, language, gesture * language interaction) and random effects (by-participant varying gesture effects) structure as the previous model. Figure 4 shows the posterior distributions of the three fixed effects coefficients taken from the logistic regression model. As can be seen, the gesture effect was far away from zero (posterior log odd mean: + 0.13), with a 95% credible interval of that firmly excludes zero (95% CrI: [+ 0.07, + 0.19]). The posterior probability of the gesture effect being of the same sign was high, *p* = 1.0 (100% of all posterior values above zero). The language effect was slightly weaker compared to our absolute magnitude analysis (posterior mean = + 0.03), with a 95% CrI that firmly includes zero [-0.03, + 0.09]. The posterior probability of the gesture effect being stronger in magnitude than the language effect was very high, *p* = 0.99. Again, there was no indication of an interaction between gesture and language (posterior mean = – 0.01; 95% Crl [-0.07, + 0.05]), indicating that both speaker groups showed similar effects.

### Individual differences

We investigated individual differences by fitting two new mixed beta-binomial regression models to the numbers generated by participants (absolute values). Each model included the fixed effect gesture (inwards versus outwards, treatment-coded) and either the ‘Brief Assessment of Gesture’ (BAG) scale or the ‘Subjective Numeracy Scale’ (SNS) scale. Each model also included the interaction between gesture and each of the scales to assess whether participants with higher/lower gesture or numeracy values show stronger or weaker effects. For random effects, we included by-participant varying slopes in the gesture effect. To aid the interpretation of coefficients, we mean-centered the BAG and SNS scales.

For the beta-binomial model of absolute magnitudes, the gesture effect was moderated by the BAG scale in a positive fashion (log odd posterior mean = 0.04), with the 95% credible interval of the interaction coefficient that barely overlapped with zero (95% CrI: [– 0.01, + 0.09]). The posterior probability of this interaction effect being of the same sign was *p* = 0.95. Thus, we found some indication that people who self-report to gesture more also show stronger gesture effects, i.e., relatively smaller numbers for inwards and relatively larger numbers for outwards movements. When looking at relative differences between consecutive numbers as opposed to the absolute magnitude of an uttered number, there also was a positive interaction between the gestural manipulation and the BAG scale (log odd coefficient: + 0.04, 95% CrI: [– 0.02, + 0.10]), although this result was linked with slightly more uncertainty, as revealed by a lower posterior probability of this effect being of the same sign, *p* = 0.9. Thus, for relative changes between consecutive numbers, we found a numerical trend for people with a higher propensity to gesture to show a stronger gesture effect on relative changes.

We fitted separate individual difference models including the interaction between gesture and SNS scale. For the absolute magnitudes, the interaction effect had the opposite sign from the BAG interaction reported above: participants with higher numeracy showed comparatively weaker gesture effects (– 0.04); the 95% credible interval of this interaction coefficient barely overlapped with zero (95% CrI: [– 0.08, + 0.01]). The posterior probability of this effect being of the same sign was *p* = 0.94. For the relative differences between consecutive numbers as opposed to absolute magnitude of individual numbers, the individual difference interaction was associated with considerably more uncertainty (– 0.03, 95% CrI: [– 0.09, + 0.03]), with a posterior probability of only *p* = 0.83 of being negative. That is, similar to the individual differences in BAG, the numeracy interaction only indicated a numerical trend.

**Fig. 5:**
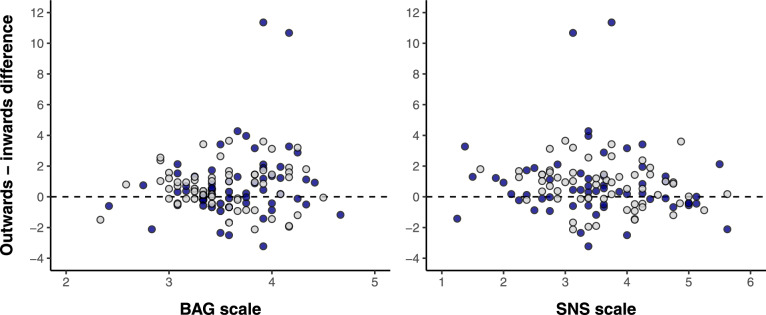
Participant average difference score (up = higher numbers for outwards trials) plotted against a) the ‘Brief Assessment of Gesture’ (BAG) scale, and b) the ‘Subjective Numeracy Scale’ (SNS); point colors indicate English (light grey) and Italian (dark blue)

Figure 5 shows scatterplots of each participant’s average difference score (outwards minus inwards) against the BAG scale and the SNS scale. As can be seen, any correlations between the individual difference measures and participants’ average gesture effects is barely visible to the naked eye, if at all. In terms of raw correlations (Spearman), we founda very weak correlation between gesture effects and the BAG scale (ϱ = 0.13), and no indication of a correlation for the SNS scale (ϱ = 0.002). These results were not strongly affected by the two participants with very large differences. If these two data points had been excluded from either the model or the correlations, conclusions would not have been altered.

### Numbers driving the effect

As discussed in Section "Absolute magnitude", we noted that on first trials, there was a high proportion of saying either 1 or 30, which were the two numbers being mentioned in the instructions. In total, when the inwards trial was the first trial, 11 participants said “1”, as opposed to only one participant who said “1” on the outwards trial. In contrast, the number “30” was mentioned never when the first trial was inwards, but 4 times when it was outwards. These figures suggest that participants were indeed somewhat primed by the instructions, but interestingly, already this priming was modulated by the gesture manipulation, with participants picking the smaller of the two numbers when the first trial was inwards, and the larger of the two numbers when the first trial was outwards.

**Fig. 6:**
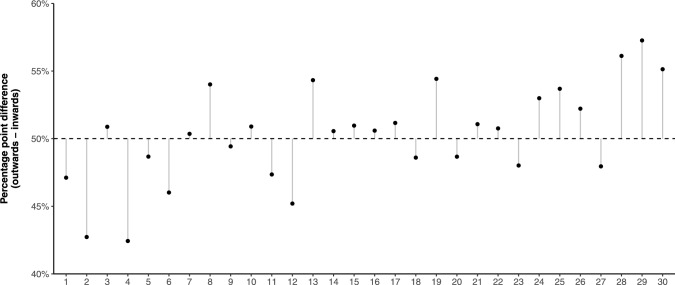
Dot plot showing the percentage difference (outwards minus inwards) for each of the numbers 1–30; vertical bars highlight whether changes are above or below the 50% midline (no difference between outwards and inwards). Y-axis range is constrained to 40–60% to make differences between numbers visible

Given this priming, we wanted to additionally explore whether the effects could have been driven by specific numbers, i.e., it is theoretically possible that a strong gesture effect only emerges on the smallest and largest numbers of our range (1–30), or that it is driven by specifically the boundary numbers 1 and 30. Figure 6 shows the average gesture effect (outwards minus inwards, averaged across all participants) separately for each number. The percentage difference correlates with numerical magnitude (Spearman’s ϱ = 0.58), which suggests that the effect we report above was not driven by only a few specific numbers. As can be seen, the effect was somewhat continuous, with less pronounced differences in the middle range, and stronger differences for more extreme magnitudes. Clearly, results were most consistent for the particularly high numbers 28, 29, and 30, all of which showed positive percentage differences (higher chance of occurring in the outwards condition). For the small numbers, there were very strong results for the even numbers 2 and 4, but less strong results for the odd numbers 1 and 3.

## Discussion

We used inwards and outwards gestures that have been attested in observational studies of numerical communication (Woodin et al., 2020) as means to investigate size-based spatial-numerical associations in random number generation. Overall, we found a reliable gesture effect: numbers were overall larger on outwards trials and smaller on inwards trials, and the number produced on an outward trial were larger than the previous one. The effect emerged under multiple different analysis approaches, but was always relatively small in effect size. While our Bayesian analysis indicated high inferential certainty in the average effect, descriptive averages, Cohen’s *d*, and the fact that in one of our analyses, the gesture effect did not exceed unexpected language group variation all speak to gestural movements biasing RNG only weakly.

Similar to other spatial-numerical associations, not every participant showed the effect. Using an approach inspired by Roth et al. (2024), participant-focused analysis indicated that only few participants reliably differed from zero in the expected direction. From this perspective, our results are not unlike what has been observed for other spatial-numerical associations, where reliable group level results do not necessarily mean that all individuals reliably show an effect (Cipora et al., 2020; Roth et al., 2024). However, we caution that this result depends on the number of trials available per participant. Our trial number (80 data points per participant) was inspired by previous RNG studies that aimed at making statements on group-level behavior; our study was not designed to measure individual consistency reliably. Descriptive averages showed that the effect had the predicted sign for 65% of all participants, and with more data per participant, it is possible that more of these individual effects would show credible intervals excluding zero if our study included more trials per participant. It is worth highlighting that there were two participants who showed very large effects that completely stood outfrom the rest. It seems plausible that these participants used an explicit response strategy where they consistently generated a larger number for outwards trials. It is important to emphasize that our results hold even if these two extreme participants are excluded.

In terms of individual differences, our results were relatively uncertain, preventing us from drawing strong conclusions. Specifically, if at all, individual differences were of very small effect size. As posterior probabilities were generally at 0.95 or lower, we conclude that we have failed to find reliable evidence for individual differences. The numerical trends we observed—people who gesture more and people with lower numeracy show slightly stronger effects—are suggestive, but we refrain from drawing any strong conclusions from them. The lack of a strong numeracy effect however is interesting from the perspective that other kinds of spatial-numerical associations have also been found not to positively correlate with mathematical proficiency. This pattern has been observed for horizontal spatial-numerical associations, which even decrease with increasing math proficiency (Cipora & Nuerk, 2013; Cipora et al., 2016, 2018a, 2018b). Consistent with this, we found that the gesture effect was diminished for participants with relatively higher numeracy. However, we caution to conclude that the numeracy individual differences we measured reflect a straightforward difference in math expertise, above and beyond general numeracy: we did not test professional mathematicians and the Subjective Numeracy Scale (SNS) indexes how much people rely on numbers in their everyday life activities; it is not informative about the specific math skill levels of participants, and does not test mathematical proficiency directly. It is worth mentioning that spatial-numerical associations in RNG tasks might also be modulated by other individual cognitive abilities, such as inhibitory control and working memory (see Baddeley, 1998; Towse & Cheshire, 2007). Future studies should further investigate these dimensions.

The gesture effect was moderated by individual differences in gesture use in a positive way, but this does not seem to be driven by cultural and linguistic factors as we failed to find a reliable difference between the Italian and English sample. Regardless of being Italian or English, participants who self-reported high values in gesture production and perception on the BAG scale showed a slightly stronger effect. One possible reason for the absence of variation at cross-cultural and cross-linguistic levels may be that the two groups did not differ in gesture frequency, but rather in the distribution of gestural functions. As previous research has shown, Italians tend to use more pragmatic gestures instead of referential gestures (see Graziano & Gullberg, 2024; see Kita, 2009 for a review). This dimension does not, however, appear to be relevant for our data.

It should also be noted that our task did not, of course, contain naturalistic co-speech gestures produced with communicative intent. Instead, our “gestures” are best viewed as being movements that serve as laboratory analogues of the numerical gestures that are produced in natural language use (Woodin et al., 2020). While we are the first to study RNG behaviors using movements that occur naturally in conversation about quantities, there was no communicative component in our task, and the task itself is still highly artificial. Importantly, Lorson et al. (2024) showed conceptually similar results to ours using the same movements in a task that involved more naturalistic linguistic stimuli: participants viewed videos of expressions like “400 people were at the protest. *Several* of them got arrested”, where outwards/inwards gestures were produced on the word *several* and reliably influenced numerical judgments. These results nicely dovetail with ours: while Lorson et al. (2024) showed that the inwards/outwards effect is found in a perception task that involved more naturalistic stimuli with a clear communicative component (see also Nicol & Patson, 2022), the current study is a production task that indexes a more automatic and low-level connection between number and space. Taken together, these studies clearly show that spatial-numerical associations triggered through gestures play a role in adult numerical cognition, and not just, as has previously been shown, in mathematical learning by children (Alibali et al., 1999; Cook et al., 2012; Goldin-Meadow, 2003; Goldin-Meadow et al., 2001; Marghetis & Núñez, 2013).

It is noteworthy that effect size is rarely discussed in studies of RNG (e.g., Hartmann et al., 2014; Loetscher et al., 2008, 2010). Winter and Matlock (2013) found a significant effect on both absolute and relative numbers in horizontal movements (Cohen’s *d* = 0.17 and *d* = 0.44; respectively) and a stronger effect in vertical movements (Cohen’s *d* = 0.43 and *d* = 0.72; respectively). The current results suggest that the effect of gesture on random number generation behavior is relatively small. This, to us, was surprising as we expected the large movements involved in our task to yield a stronger effect than, for example, the concomitant head movements that were used in previous tasks. This expectation stems from the fact that such head movements are relatively small movements. Moreover, in contrast to the movements we investigated here, which are attested in spontaneous discourse (Woodin et al., 2020), there is no evidence up to this point that speakers move their head position in relation to the numbers they talk about. As we are the first to explicitly emphasize the weak nature of this effect, we caution that future studies of RNG should give more consideration to effect size. In the literature on embodied cognition in numerical thinking and other domains, there is much interest in the functional relevance of low-level perceptual phenomena on high-level cognitive tasks (see among others, Willems et al., 2011; Vukovic et al., 2017, and for a discussion see Ostarek & Huettig, 2019). Nevertheless, many experimental results are reported in a categorical manner, i.e., researchers emphasize *that* there is an embodied effect. *How much* embodied factors matter in terms of effect size is often not as actively incorporated into theorizing. For the phenomenon we investigated here, it is clear that while spatial-numerical associations are actively triggered by gestures and do influence number production in RNG, they only do so very little. That is, we have conclusively shown that gestures matter, but they clearly do not matter by a lot: our gestures nudged production without dominating the picture. At least when considering the specific task here, this puts constraints on how influential embodied effects are taken to be. Future work needs to take effect size more seriously, moving beyond demonstrations *that* space matters to discussion of *how much* it matters, to advance theorizing in this field.

## Conclusion

We investigated how inward and outward gestures, observed in natural numerical discourse, influence size-based spatial-numerical associations during random number generation. Our findings evidenced a reliable gesture effect at the group level: numbers generated with outward gestures were larger than those produced with inward gestures, both in terms of absolute magnitude and relative to the previous number. This effect was small in effect size. We found no compelling evidence for cultural or individual differences. There was no evidence that the effect varied between Italian and English participants, and only suggestive evidence that participants who self-reported to gesture more frequently and who self-reported to have lower numeracy had a slightly stronger effect. These results show that spatial gestures influence numerical representation, but they also suggest limits of the functional relevance of spatial-numerical associations.

## Data Availability

All data and analysis code is available on the following Open Science Framework repository: https://osf.io/9esjz/.

## Footnotes

1 https://www.imusic-school.com/en/tools/online-metronome/.

2 Although we prefer to model the underlying data-generating processes directly to ascertain an explicitly generative model, it is worth pointing out that simple significance tests also confirm our main hypothesis. For example, paired t-tests (*t*(119) = 4.23, *p* < 0.0001) or Wilcox tests (*V* = 1998.5, *p* < 0.0001) on subject means for outwards and inwards trial yield ‘significance’, and so does an exact binomial test on a participant’s sign of the effect (78 out of 120 participant means have the correct sign, probability of success = 0.65, *p* = 0.0013).
